# Identification of novel inhibitors for SARS-CoV-2 as therapeutic options using machine learning-based virtual screening, molecular docking and MD simulation

**DOI:** 10.3389/fmolb.2023.1060076

**Published:** 2023-03-07

**Authors:** Abdus Samad, Amar Ajmal, Arif Mahmood, Beenish Khurshid, Ping Li, Syed Mansoor Jan, Ashfaq Ur Rehman, Pei He, Ashraf N. Abdalla, Muhammad Umair, Junjian Hu, Abdul Wadood

**Affiliations:** ^1^ Department of Biochemistry, Abdul Wali Khan University, Mardan, KPK, Pakistan; ^2^ Center for Medical Genetics and Hunan Key Laboratory of Medical Genetics, School of Life Sciences, Central South University, Changsha, Hunan, China; ^3^ Institute of Molecular Precision Medicine, Xiangya Hospital, Central South University, Changsha, Hunan, China; ^4^ Institutes of Biomedical Sciences, Shanxi university, Taiyuan, China; ^5^ Department of Molecular Biology and Biochemistry, University of California, Irvine, Irvine, CA, United States; ^6^ Department of Obstetrics and Gynecology, Nanfang Hospital, Southern Medical University, Guangzhou, China; ^7^ Department of Pharmacology and Toxicology, College of Pharmacy, Umm Al-Qura University, Makkah, Saudi Arabia; ^8^ Department of Life Sciences, School of Science, University of Management and Technology (UMT), Lahore, Pakistan; ^9^ Department of Central Laboratory, SSL Central Hospital of Dongguan City, Affiliated Dongguan Shilong People’s Hospital of Southern Medical University, Dongguan, China

**Keywords:** SARS-CoV-2, COVID, 19, machine learning, molecular docking, MD simulation, Corona virus

## Abstract

The new coronavirus SARS-COV-2, which emerged in late 2019 from Wuhan city of China was regarded as causing agent of the COVID-19 pandemic. The primary protease which is also known by various synonymous i.e., main protease, 3-Chymotrypsin-like protease (3CL^PRO^) has a vital role in the replication of the virus, which can be used as a potential drug target. The current study aimed to identify novel phytochemical therapeutics for 3CL^PRO^ by machine learning-based virtual screening. A total of 4,000 phytochemicals were collected from deep literature surveys and various other sources. The 2D structures of these phytochemicals were retrieved from the PubChem database, and with the use of a molecular operating environment, 2D descriptors were calculated. Machine learning-based virtual screening was performed to predict the active phytochemicals against the SARS-CoV-2 3CL^PRO^. Random forest achieved 98% accuracy on the train and test set among the different machine learning algorithms. Random forest model was used to screen 4,000 phytochemicals which leads to the identification of 26 inhibitors against the 3CL^PRO^. These hits were then docked into the active site of 3CL^PRO^. Based on docking scores and protein-ligand interactions, MD simulations have been performed using 100 ns for the top 5 novel inhibitors, ivermectin, and the APO state of 3CL^PRO^. The post-dynamic analysis i.e,. Root means square deviation (RMSD), Root mean square fluctuation analysis (RMSF), and MM-GBSA analysis reveal that our newly identified phytochemicals form significant interactions in the binding pocket of 3CL^PRO^ and form stable complexes, indicating that these phytochemicals could be used as potential antagonists for SARS-COV-2.

## 1 Introduction

SARS-CoV-2 is a single-strand RNA, positive sense, and enveloped beta coronavirus that causes respiratory, nervous, hepatic, and human gastrointestinal diseases (Tahir ul Qamar et al., 2020) Wuhan, a city in China, was the first city to be infected by the virus in December 2019 (Zhu et al., 2019; Zhou et al., 2020). COVID-19 outbreak was declared a pandemic by the World Health Organization (WHO). The infection spreads rapidly across the World. By the end of October 2020, more than 60 million people were infected by COVID-19, resulting in more than 1.4 million fatalities. The number of patients and fatalities was rising, posing a major threat to global health. High temperature, coughing, shortness of breath, and severe cases that can result in renal failure and even death are some of the symptoms of COVID-19 infections (Rothan and Byrareddy, 2020; Asif et al., 2022), until now, there is no effective treatment available yet.

SARS-CoV-2 is a member of the beta coronavirus family (Marty and Jones, 2020), usually, during the process of transcription, beta coronaviruses produce an 800 kDa polypeptide (Xu et al., 2020). The genome of the novel SARS-CoV-2 was recently sequenced and compared with those of existing coronaviruses (CoVs) by Wu et al. who identified that the novel SARS-CoV-2 belonged to the β-CoVs, which were initially discovered in bats and have now evolved to infect humans (Wu et al., 2020a). The SARS-CoV-2 genome is approximately 30 kb in size and is comprised of at least six open reading frames (ORFs) which are responsible for encoding the whole proteome of the virus. The coding RNA contains the structural, non-structural protein (Nsps) coding regions and the accessory protein-coding region (Durojaiye et al., 2020). The genes on the 3′-terminus encode the four structural proteins including the spike protein, membrane, envelope, nucleocapsid, and many accessory proteins. The membrane, envelope, and nucleocapsid protein protect the virus before entering the host cell. The Spike protein of SARS-CoV-2 comprises S1 and S2 subunits. The receptor-binding domain is a part of the S1 subunit that plays role in the attachment of the virus with the receptor while viral cell membrane fusion is mediated by the S2 subunit, thus facilitating the virus entry (Alanagreh et al., 2020; Jackson et al., 2021). The SARS-CoV-2 virus’s replication and ability to spread are facilitated by numerous crucial proteins and enzymes. Two essential proteases, main protease (3CL^PRO^) and papain-like protease (PLpro) are necessary for viral replication (Huang et al., 2020; Mouffouk et al., 2021). The non-structural proteins nsp1, nsp2, and nsp3 are known to be cleaved by PLpro, while the remaining 13 are cleaved by 3CL^PRO^ (Klemm et al., 2020). The 3CL^PRO^ cleaves polypeptide sequences after a glutamine residue, making it a perfect drug target as no human host-cell proteases with this cleavage specificity are identified (Hilgenfeld and Hilgenfeld, 2014; Ullrich and Nitsche, 2020).

The structure of the 3CL^PRO^ comprises three important domains, domain-I ranges from 8–101, while domains-II corresponds to position 102–184, followed by the connecting loop from 185–200, which links domain-II and domain-III, domain-III has a total number of 103 residues which lies after the connecting loop from 201–303 (Wu et al., 2020b). Furthermore, the His-41 and Cys-145 form an essential catalytic dyad (Kneller et al., 2020). Small compounds that target conserved viral proteases, such as the major protease, may thus be able to inhibit crucial phases of the SARS-CoV-2 life cycle while causing few adverse effects (Mengist et al., 2021). Approved drugs have been developed for viral infections such as those caused by Hepatitis C virus and human immunodeficiency virus for the target’s serine proteases and aspartyl protease respectively which employ that viral proteases are well-established therapeutic targets (Agbowuro et al., 2018). Antiviral drugs are required in this situation to prevent infection in high-risk populations as well as to treat infected patients. Developing inhibitors that stop coronavirus replication can recover millions of people globally. In the clinical investigations, efforts to repurpose the majority of approved drugs have discovered several promising candidates (such as remdesivir and hydroxychloroquine) but these drugs had little to no effect on mortality and the duration of hospital stay (Luttens et al., 2022). Hence, it is crucial to find new drug candidates that would target various SARS-CoV-2 proteins for increased COVID-19 therapeutic effectiveness (Elmaaty et al., 2022). Despite the significant cost and time required for the development of the new drug, clinical trials only yield a 13 percent success rate, while in 40%–60% of cases, drugs failed to reach the market because of the lack of optimum pharmacokinetic properties (Gurung et al., 2021).

The use of computer-aided drug discovery (CADD) tools helps to accelerate the process of drug discovery and to reduce costs (Macalino et al., 2015) In addition, the advent of supercomputing facilities, algorithms, and tools has enhanced lead identification in pharmaceutical research (Macalino et al., 2018). Artificial intelligence (AI) and machine learning approaches have substantially assisted the analysis of pharmaceutical-related large data in the drug discovery process (Floresta et al., 2022). Furthermore, the structure-based drug development method is specific and successful in identifying lead compounds and optimizing them, and it has aided in the understanding of disease at the molecular level (Yang et al., 2022). In the current study, we employed different machine learning (ML) models for the virtual screening of phytochemicals against the 3CL^PRO^ drug target in SARS-CoV-2. The active hits obtained from ML-based were passed through an electronic filter called PAINS filter and their ADMET (absorption, distribution, metabolism, excretion, and toxicity) properties were examined. The active phytochemicals that passed through the PAINS filter and have enhanced properties were further considered for the molecular docking analysis. Furthermore, the stability and binding energy of these compounds in the active site of 3CL^PRO^ were investigated by 100 ns of MD simulations. Based on our findings we suggest these phytochemicals as potent inhibitors of SARS-CoV-2 3CL^PRO^, *In vitro* evaluation of these compounds, is essential for the understanding of their action and mechanism to cope with such a pandemic.

## 2 Methodology

**FIGURE 1:**
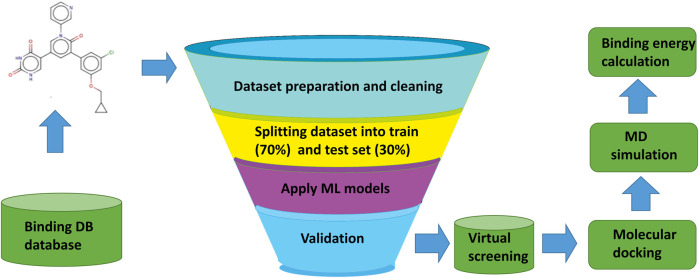
Overall workflow of machine learning based virtual screening, molecular docking, and MD simulation study for 3CL^PRO^ (3C like protease) a validated drug target in SARS-CoV-2.

The overall workflow of the current study, from the collection and preparation of the dataset of active and inactive compounds, screening of compounds, molecular docking, and binding energy calculations are represented in Figure 1.

### 2.1 Preparing and cleaning the dataset

From the binding DB database (Sandhu et al., 2022) a total of 101 molecules were retrieved for 3CL^PRO^ (3C like protease) a drug target in SARS-CoV-2. A total of 500 decoys molecules, which are considered to be inactive, were generated using the DUDE database (Mysinger et al., 2012) Out of the total 601 compounds (Supplementary Table S1), 101 compounds from the binding DB database were labeled as “1” active, and the 500 decoys were labeled as “0” inactive. The Pandas library of python was used for data preprocessing and data cleaning (Santos et al., 2020). The dataset was split into train set (70%) and a test set (30%).

### 2.2 Features calculation

The 2D features of all the compounds were calculated using MOE (2016) software (Wadood et al., 2022a). Total 206 features were calculated. Feature with 0 or null values were removed from the dataset to reduce the computation time.

### 2.3 Principal component analysis (PCA)

The dataset was uploaded to iRaPCA v1.0 implemented in the LideB tool in CSV format. The optimum subsets of descriptors were selected from the dataset. The dimensionality was reduced by performing the PCA. The process is based on the principle of feature bagging (Prada Gori et al., 2022). The conventional feature extraction and data representation method used extensively in the fields of pattern recognition is principal component analysis (PCA), generally called as Karhunen-Loeve expansion. PCA is a method for reducing high-dimension data to low-dimension while preserving the majority of the relevant data. The main benefits of PCA are its low noise sensitivity, lower capacity and memory requirements, and increased performance (Karamizadeh et al., 2013).

### 2.4 Machine learning models

#### 2.4.1 K nearest neighbor model

The distance-based classification algorithm is called k-Nearest Neighbors (kNN), which is an effective and simple machine learning algorithm widely used for the classification of active and inactive compounds in the dataset (Wadood et al., 2022b)*.* The accuracy of the kNN model depends entirely on the quality of the data. One of the most difficult parts of KNN is figuring out how many neighbors to consider. The KNN can be used for both classification and regression (Sarker, 2021a).

#### 2.4.2 Support vector machine (SVM)

SVM is generally used for the classification of data. SVM is based on the principle of calculating margins between two classes. This classifier reduced the error by drawing the margins in a manner where the distance between the margin and the classes is as large, as possible (Noreen et al., 2016). The SVM classifier depends on the kernel function and is more effective for high-dimensional data classification. When the dataset contains additional noise, such as overlapping target classes, SVM does not perform effectively (Sarker, 2021b).

#### 2.4.3 Random forest

Random forest (RF) is an ensemble algorithm made up of several decision trees, similar to how a forest is made up of many trees (Breiman, 2001). To train, the decision trees of a random forest various subsets of the training dataset are used. To classify a new sample, the sample’s input vector must be passed down from each decision tree of the forest. This algorithm classifies the data using majority voting. In terms of performance, RF performs better than a decision tree. For huge datasets, it works effectively. The classifier also calculates which variables or attributes are most significant in the classification (Ul Hassan et al., 2018). The sklearn library of python was used for developing the three machine learning models.

#### 2.4.4 Naïve bayes

The naive Bayesian algorithm is based on the Bayes theorem and is a reliable classification method. A data set can be classified by NB classifier assuming that every feature contributes equally and independently (Patel et al., 2020). In this study, the NB classifier was built using python v.3.9.

#### 2.4.5 Cross-validation and performance evaluation

We used 10-fold cross-validation in this study. The performance of the models was accessed by using several statistical parameters including accuracy, sensitivity, specificity, F1 score, MCC (Ahmad et al., 2021).

### 2.5 Virtual screening of the asian phytochemicals

A list of Asian plants with notable therapeutic properties was compiled, and then a thorough literature search was performed to determine the phytochemical contents. The compound collection was carried out using Google Scholar, PubMed, MEDLINE, and other web-based resources. A total of 4,000 phytochemical libraries was generated, and the 2D structure of these phytochemicals was retrieved from the PubChem database. Before adding to the library all these phytochemicals were cleaned and energy minimized using the mmff94 force field.

### 2.6 PAIN filter

Pre-filtering large databases using appropriate molecular properties is a typical approach to reduce computing and get rid of unwanted compounds (Baell and Holloway, 2010). All the active hits were filtered by an online tool PAINS (Wadood et al., 2022c) and only those compounds were further selected for docking that was passed from the PAINS filters.

### 2.7. Molecular docking study

#### 2.7.1 Preparation and validation of target protein

The 3D structure of SARS-CoV-2 3CL^PRO^ (PDB ID: 6LU7; Resolution: 2.16 Å; Organism: SARS-CoV-2; Method: X-ray diffraction) was downloaded from the RCBS Protein Data Bank (Hatada et al., 2020). There are two chains in the crystal structure: A and C. The macromolecule chain A was chosen as the target receptor. Pymol was used to remove water molecules and heteroatoms from the protein structure (Janson et al., 2017). The structure was then energy minimized using ff14sb implemented in the molecular operating environment (MOE) (Ashraf et al., 2021). The PROCHECK (Laskowski et al., 1996) and ERRAT (Colovos and Yeates, 1993) tools from the Structural Analysis and Verification Server (SAVES) (http://nihserver.mbi.ucla.edu/SAVES) were used to validate the crystal structure. The stereo chemical quality of the protein structure was evaluated using PROCHECK.

#### 2.7.2 Molecular docking protocol

All the phytochemicals predicted as active by the machine learning method were docked into the active site of a SARS-CoV-2 3CL^PRO^ for molecular interaction studies. The crystal structure of the SARs-CoV-2 3CL^PRO^ (PDB ID: 6LU7) is complex with an N3 inhibitor was retrieved from the PDB database. The Inhibitor N3 is linked to the protease at site one of this crystal structure, which contains five cavities for ligand binding (Das et al., 2021). We used the N3 binding site (site 1) for virtual screening of these phytochemicals’ library. For the molecular docking study, MOE v2016 was used to run a docking protocol using rigid and ligand-based docking parameters. The Triangular Matching docking method (default) was used and a total of ten poses were generated for each Phytochemical (Thuy et al., 2020). The best S score hits against 3CL^PRO^ were considered for the molecular interactions study and their 3D images were generated by PyMol software. A total of 05 top-ranked compounds were shortlisted for further molecular dynamic simulations analysis based on the docking score. These phytochemicals are structurally diverse, effective, and new inhibitors for the main protease, according to the docking score, binding mode, and visual ligand interaction.

### 2.8 MD simulations

Molecular dynamics simulation is a powerful tool to understand the dynamics and interaction behavior of the reference complex and the selected top hits were used. The ff14SB protein force field in Amber 20 package was employed (Salomon-Ferrer et al., 2013a). For solvation of each system, the tip3p water model with box dimension 8.0 was used. All of the systems were adequately solvated and neutralized by adding four Na + ions to counterbalance the charges on the systems. Afterward, energy minimization for 6,000 steps of neutralized complexes was carried out using the steepest descent minimization algorithm, then progressively heated to 300 K before equilibrating density for 2 ns with weak constraints. The whole system was equilibrated at constant pressure for another 2 ns. A Langevin thermostat was used to control the temperature 300 K. Further, a 100-ns MD was performed on the equilibrated systems. For long-range electrostatic interactions, Particle Mesh Ewald (PME) algorithm was used (Darden et al., 1998). For covalent bonds including hydrogen, the SHAKE algorithm was utilized. Finally, a 100 ns MD simulation of all equilibrated complexes at constant pressure and temperature was carried out by using PMEMD.cuda (Salomon-Ferrer et al., 2013b).

### 2.9 DCCM

The dynamic cross-correlation analysis is useful for explaining the correlation among the residues represented by a three-dimensional matrix. The cross-correlation was calculated by the formula (Junaid et al., 2018)

Cij=∆ri.∆rj∆ri2∆rj21/2 (1)

Where the mean position of ith and jth atom is represented by ∆r_i_, ∆r_j_ respectively. Where the angular brackets are used to measure the average time of the entire trajectories produced as a result of MD simulations. Positive Correlated movement such as movement in the same direction is represented by the positive value of Cij, while the negative value of Cij reflects strong anti-correlation movements between the residues. Cpptraj was used to perform DCCM analysis while origin 2021 was used for graphical representations (Perez-Lemus et al., 2022).

### 2.10 Binding affinity calculations

To study the interaction between protein and ligand, binding free energy calculations play an important role. Using MMPBSA. PY script, the binding free energy between main protease and phytochemicals inhibitors was calculated (Gul et al., 2021). The following equation was used to calculate the free energy of each energy term:

$$∆G_{bind}=∆G_{complex}-\left[∆G_{receptor}+∆G_{ligand}\right]$$ (2)

In the equation, ${∆G}_{bind}$ represents the total binding free energy, $∆G_{\mathrm{complex}}$ denotes the free energy of complex, $∆G_{\mathrm{receptor}}$ and $∆G_{\mathrm{ligand}}$ represents the free energy of receptor protein and ligand respectively. The following equation was used to calculate the individual free energy of complex, protein and ligand.

$$G_{X}=E_{MM}\;–\;\left(TS\right)+\left(G_{solvation}\right)$$ (3)

Where x denotes complex, protein or ligand, the average molecular mechanic potential in a vacuum is given by E_MM_, the entropic and temperature contribution is represented by TS, while the free energy of the solvation is given by $G_{solvation}$ .

## 3 Results

### 3.1 Data preparation

A total of 101 molecules were retrieved from the binding databank database for 3CL^PRO^ a drug target in SARS-CoV-2. The 101, molecules were categorized as active molecules. The remaining 500 decoys molecules were labeled as inactive. The dataset was split into a train set (70%) and test set (30%). Out of the total 601 molecules, the train set contains 420 compounds while the test set contains 181 compounds. The active and inactive compounds of the train and test set are present in Table 1.

**TABLE 1:** Train and test set used in the study.

Dataset	Inhibitors	Non-inhibitors	Total
Train	32	388	420
Test	33	148	181

### 3.2 Principle component analysis

**FIGURE 2:**
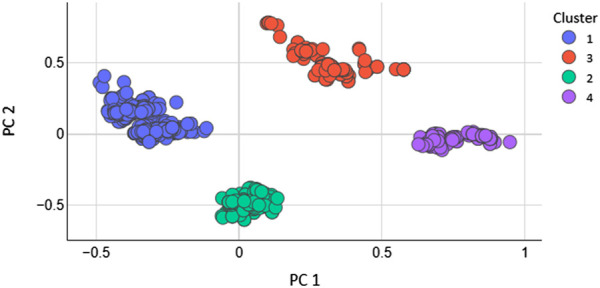
Scatter plot of PC1 vs. PC2 for subset 23 and K 4.

Total 208 2D features were calculated with the help of MOE software. The feature with 0 values were removed. As, not every extracted feature will necessarily depict the optimal properties of molecules. Therefore, optimization was carried out to get rid of duplication. Additionally, after applying the PCA the features that have higher significance were used to train the models (Araki et al., 2016). After applying PCA the data size (N) of the dataset was decreased. To evaluate how the PCA manages to maintain the dominant properties throughout the classification tasks. The models were generated by using the entire dataset without optimum features selection and the performance of the models was evaluated. It was found that the accuracy of SVM was very low as 61% and the MCC was 0.27. The accuracy of KNN model was 70% with an MCC value of 0.58 while the accuracy of RF model was 90% with an MCC value of 0.78. However, after the optimum features selection and the reduction of the dimension of the dataset the performance of all the models was greatly improved. If we want to reveal variance in a dataset having x-y coordinates, PCA finds a new coordinate system in which x, y coordinates have a different value. A new coordinate is created by the axes PC1 and PC2. These are combinations of the x-y coordinate system. Figure 2 shows the scatter plot of PC1 vs. PC2 for K = 4.

#### 3.2.1 Chemical space and diversity analysis

**FIGURE 3:**
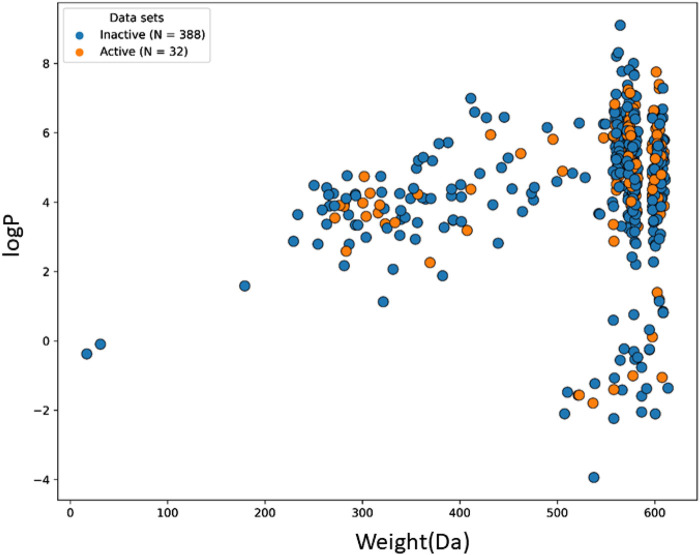
The chemical space and diversity distribution of the train set. The molecular weight and LogP define the chemical space.

**FIGURE 4:**
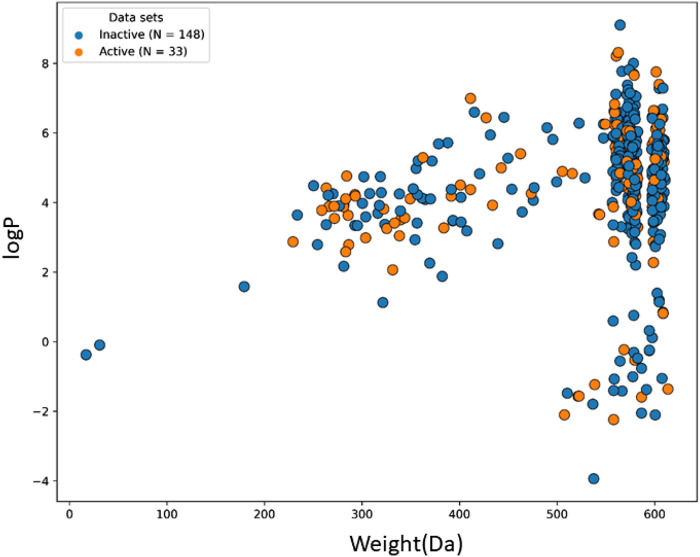
The chemical space and diversity distribution of the test set. The molecular weight and LogP define the chemical space.

The machine learning model’s accuracy is predicted by the chemical diversity of the samples from the training and test sets. The applicability of machine learning models is restricted by a small number of samples. As a result, in the present study’s physiochemical distribution analysis of the training set and test set for the molecular weight (MW) and LogP was conducted (Figures 3, 4) with MW ranging from 50 to 800 Da and LogP ranging from −2 to 15.

### 3.3 Models generation and validation

Machine learning algorithms such as kNN, SVM, RF and GNB were used for the classification of the active inhibitors against 3CL^PRO^. The sklearn library of python was used for developing the models. All the models were trained on the dataset downloaded from the binding DB database. The performance of the models was accessed by using a number of statistical parameters including accuracy, sensitivity, specificity, and MCC. Table 2 displays the over-all performance of the models on the train set while Table 3 displays the performance of all the models on the test set.

**TABLE 2:** Overall performance of machine learning models on the train set.

Model	Accuracy (%)	Sensitivity	Specificity	MCC
KNN	97	0.88	0.99	0.91
SVM	98	0.90	0.99	0.93
RF	98	0.97	0.99	0.96
GNB	94	0.83	0.96	0.79

**TABLE 3:** Performance of models on the test set.

Model	Accuracy (%)	Sensitivity	Specificity	MCC
KNN	94	0.75	0.98	0.78
SVM	96	0.82	0.99	0.87
RF	98	0.95	0.99	0.95
GNB	96	0.86	0.98	0.85

**FIGURE 5:**
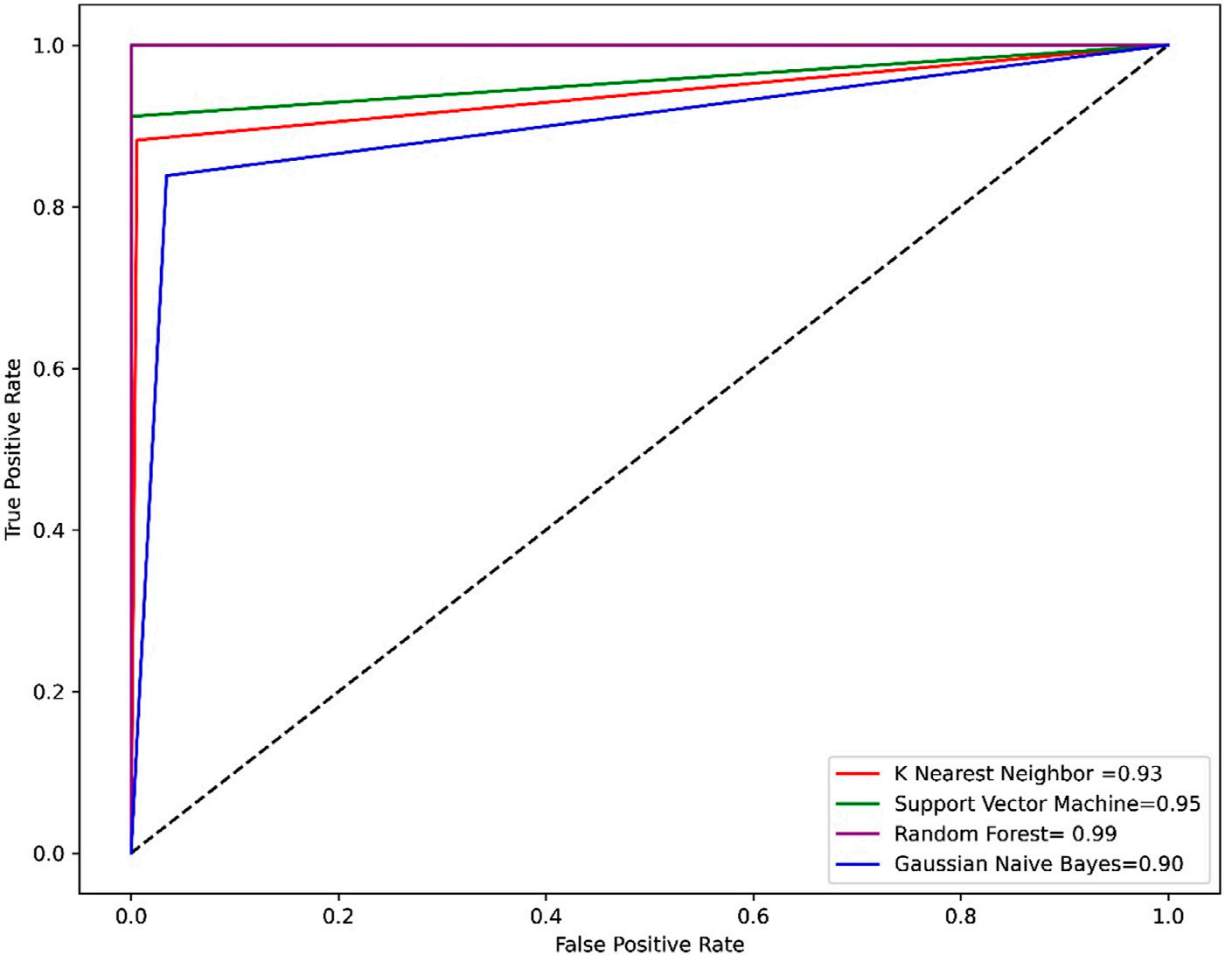
The ROC-AUC curve of all the models on the train set. The graph shows the TP against FP rate.

**FIGURE 6:**
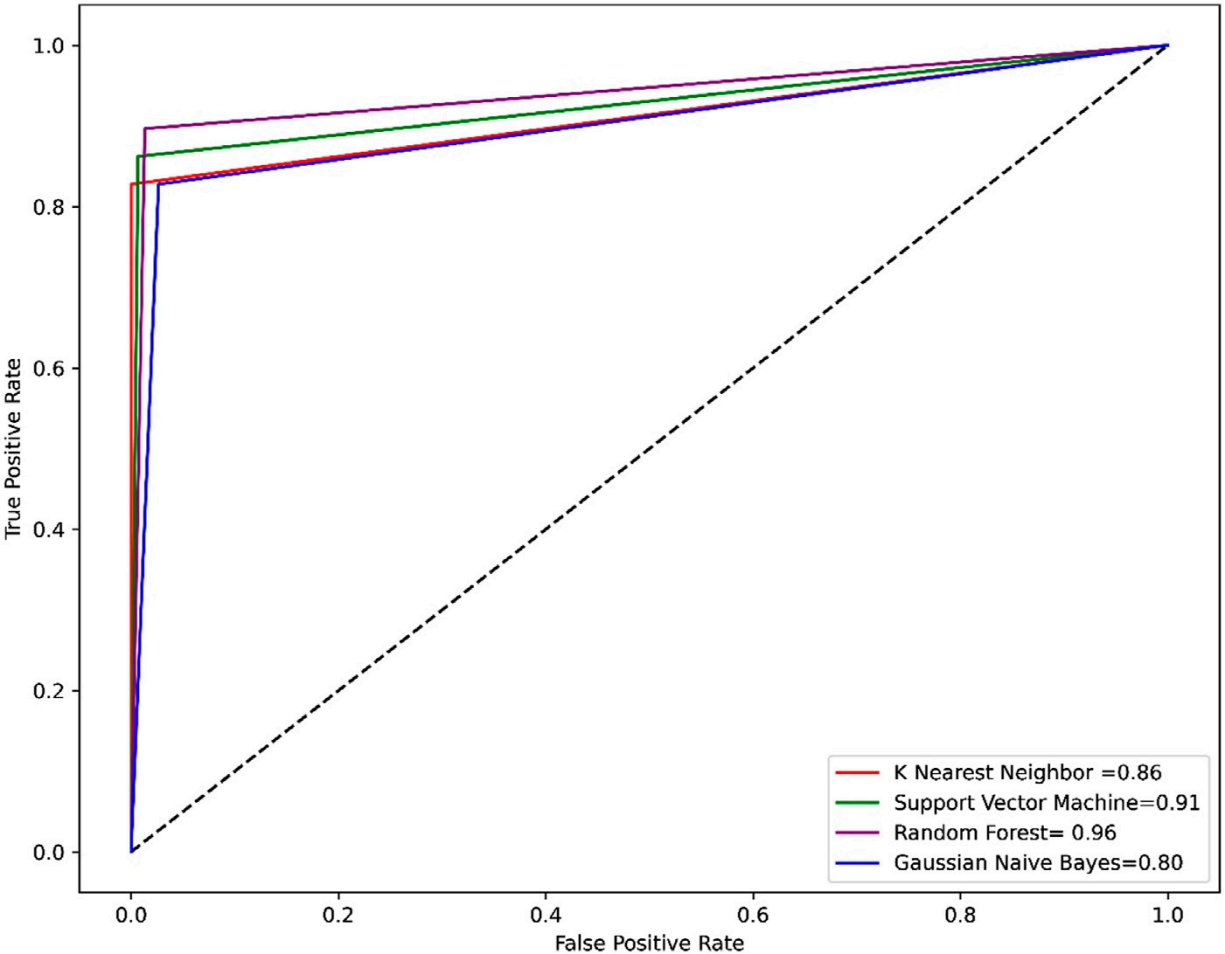
ROC-AUC curve of all the models on the test set. The graph shows the TP against FP rate.

Compared to other machine learning models random forest model achieved better accuracy and MCC value. Model performance is proportional to the area under the curve (AUC). RF has the highest AUC, followed by SVM on the training and test set Figures 5, 6. Further, we used RF model to classify the active phytochemicals against the 3CL^PRO^ enzyme. Out of 4,000 phytochemicals, a total of 26 phytochemicals were predicted as active against the 3CL^PRO^.

### 3.4 PAIN filter

Using the online PAINS tool all the hits were examined for their ADMET (absorption, distribution, metabolism, excretion, and toxicity) (Supplementary Table S2) properties. A total of seven compounds were passed from the PAINS filter and only two compounds were out of the limit. The structure of compound along with IUPAC name of the compounds passed from the PAIN filter are given in Table 4.

**TABLE 4:** PubChem ID of the compound, IUPAC name of compound and the PAIN filter result of the compounds.

Compound ID	Structure	IUPAC name	PAINS filter
91895373 (Compound 1)	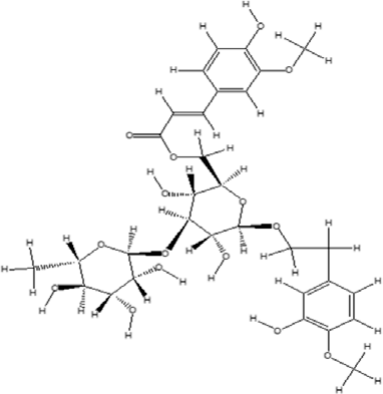	[(2*R*,3*R*,4*S*,5*R*,6*R*)-3,5-dihydroxy-6-[2-(3-hydroxy-4-methoxyphenyl) ethoxy]-4-[(2*S*,3*R*,4*R*,5*R*,6*S*)-3,4,5-trihydroxy-6-methyloxan-2-yl] oxyoxan-2-yl] methyl (*E*)-3-(4-hydroxy-3-methoxyphenyl) prop-2-enoate	Passed
10606127 (Compound 2)	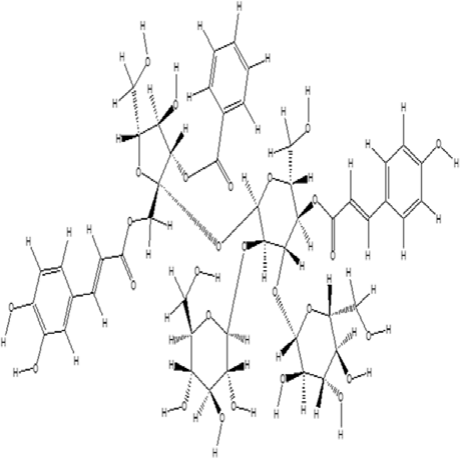	[(2*S*,3*S*,4*R*,5*R*)-2-[[(*E*)-3-(3,4-dihydroxyphenyl) prop-2-enoyl] oxymethyl]-4-hydroxy-5-(hydroxymethyl)-2-[(2*R*,3*R*,4*S*,5*R*,6*R*)-6-(hydroxymethyl)-5-[(*E*)-3-(4-hydroxyphenyl) prop-2-enoyl] oxy-3,4-bis[[(2*S*,3*R*,4*S*,5*S*,6*R*)-3,4,5-trihydroxy-6-(hydroxymethyl) oxan-2-yl] oxy] oxan-2-yl] oxyoxolan-3-yl] benzoate	Passed
5318857 (Compound 3)	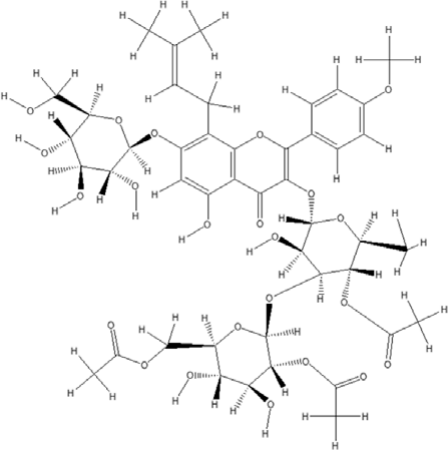	(5R,10S,13R,16R,19S)-10-[(4S,5S)-4-[(4S,6R)-4,5-dihydroxy-6-(hydroxymethyl)-3-[(2S,3R,5S)-3,4,5-trihydroxy-6-(hydroxymethyl) oxan-2-yl] oxyoxan-2-yl] oxy-3,5-dihydroxyoxan-2-yl] oxy-16,19-dihydroxy-4,5,9,9,13,19,20-heptamethyl-21-oxahexacyclo [18.2.2.01,18.04,17.05,14.08,13] tetracos-17-en-22-one	Passed
457885 (Compound 4)	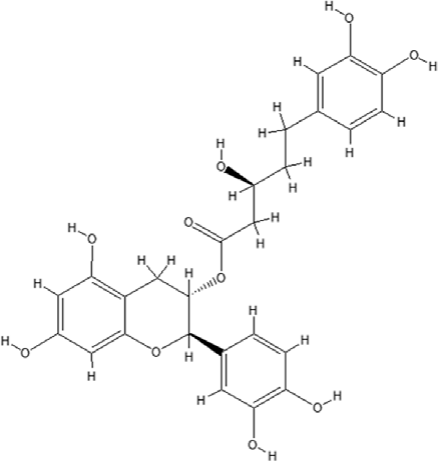	[(2R,3S)-2-(3,4-dihydroxyphenyl)-5,7-dihydroxy-3,4-dihydro-2H-chromen-3-yl] (3S)-5-(3,4-dihydroxyphenyl)-3-hydroxypentanoate	Passed
44256914 (Compound 5)	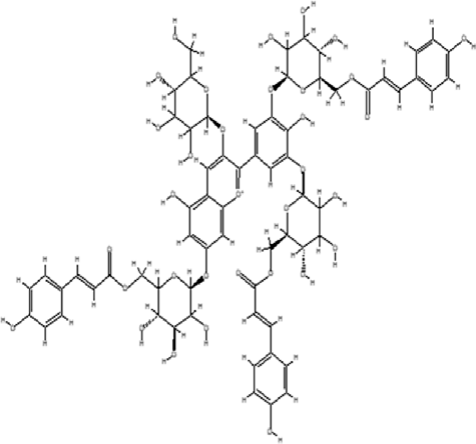	[(3S,4S,6S)-3,4,5-trihydroxy-6-[5-hydroxy-2-[4-hydroxy-3,5-bis[[(2S,5S,6R)-3,4,5-trihydroxy-6-[[(E)-3-(4-hydroxyphenyl) prop-2-enoyl] ox methyl] oxan-2-yl] oxy] phenyl]-3-[(2S,5S)-3,4,5-trihydroxy-6-(hydroxymethyl) oxan-2-yl] oxychromenylium-7-yl] oxyoxan-2-yl] methyl (E)-3-(4-hydroxyphenyl) prop-2-enoate	Passed
6321424 (Reference compound)	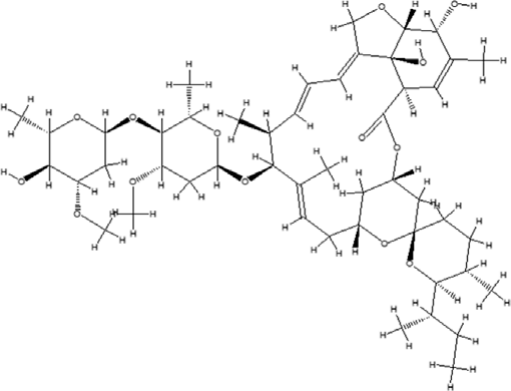	(1R,4S,5′S,6R,6′R,8R,10E,12S,13S,14E,16E,20R,21R,24S)-6'-[(2S)-butan-2-yl]-21,24-dihydroxy-12-[(2R,4S,5S,6S)-5-[(2S,4S,5S,6S)-5-hydroxy-4-methoxy-6-methyloxan-2-yl]oxy-4-methoxy-6-methyloxan-2-yl]oxy-5′,11,13,22-tetramethylspiro[3,7,19-trioxatetracyclo[15.6.1.14,8.020,24]pentacosa-10,14,16,22-tetraene-6,2′-oxane]-2-one	Passed

### 3.5 Molecular docking analysis

**FIGURE 7:**
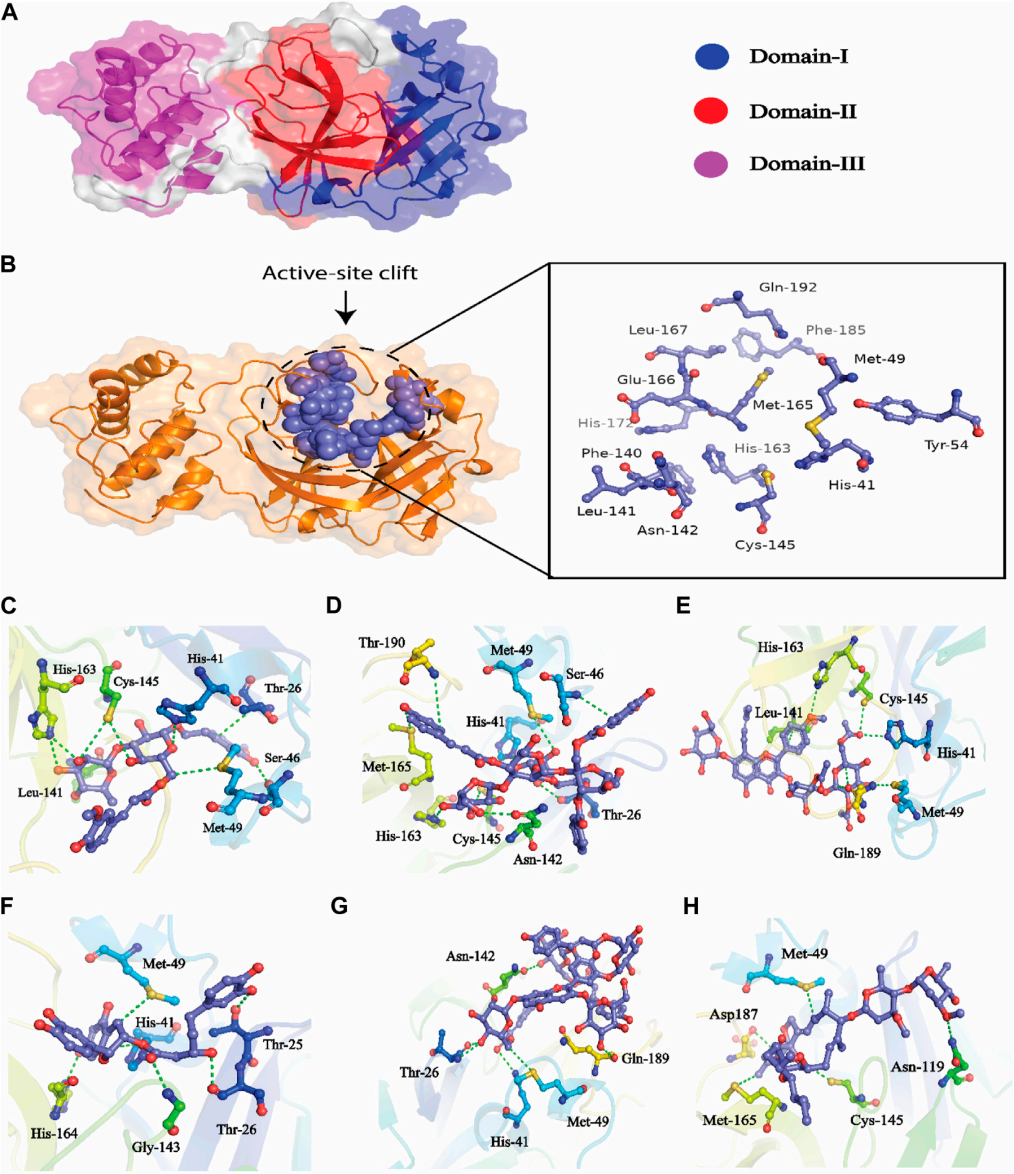
**(A)** All the three domains of 3CL^PRO^, **(B)** active site of the main protease and **(C)** indicates the interaction of Compound 1 in the active site of 3CL^PRO^, **(D)** represents the 3D interactions of Compound 2, **(E)** indicates the 3D interaction of Compound 3, **(F)** indicates interactions of Compound 4, **(G)** indicates the interaction of Compound 5, **(H)** indicating the three-dimensional interactions of the Control compounds (Ivermectin) with the 3CL^PRO^.

The hits obtained from ML based virtual screening were further used for molecular docking study. The crystal structure of the SARs-CoV-2 3CL^PRO^ (PDB ID: 6LU7) is complex with an N3 inhibitor was retrieved from the PDB database. PROCHECK tool was used to assess the 3D model’s quality of the 3CL^PRO^ structure using the Ramachandran plot (Figure S2a). The Ramachandran plot for the 3CL^PRO^ structure showed that 84.5% of residues were in the most favored region, while 14.3% were in the additional allowed region, 1.1% residues were in the generously allowed region and 0% residues were in the disallowed region demonstrating the high quality of the 3CL^PRO^ structure. For non-bonded atomic interactions, ERRAT is also known as the “overall quality factor,” with higher scores reflecting the high quality. For a high-quality model, the accepted range is > 50 (Messaoudi et al., 2013) The ERRAT server predicted an overall quality factor of 85.90 for the 3CL^PRO^ structure used in our study (Figure S2b). The interaction of top hits and the reference compound were analyzed, and it was found that all of the compounds have potent inhibitory effects on 3CL^PRO^. In order to study the interactions of these compounds in detail, the 3D visualization and compound interaction analysis was carried out. According to the interaction details Table 5, Compound 1 has stronger interaction among all of the docked compounds, it has 04 hydrogen bond donor interactions with the active site residues i.e., CYS145, SER46, and MET49, with four hydrogen bond acceptor interactions with HIS41, LEU141, and HIS163, along with one π-stacking interaction with residue THR25 with the docking score of −12.0321. Similarly, the interactions details of Compound 2 reveal that it shares five hydrogen bond donor interactions with key active site residues of the main protease i.e., THR26, MET49, ASN142, CYS145, and MET165, and two π-H interactions with residues with SER46 and THR90 respectively. The interaction table indicates that Compound 3 forms 6 hydrogen bond interactions with His41, Met49, Cys145, His163, and Gln189, and one π-H interaction with Leu 141. Compound 4 shows 04 hydrogen bond donor interactions with the catalytic residues i.e., Thr 25, Thr26, Met49, and His164, and one hydrogen bond acceptor interaction with Gly143, with one π−π interaction with residue His41. Afterward, we analyzed the interaction of Compound 5, the finding of interaction analysis indicates that Compound 5 interacts *via* four hydrogen bond donor interactions with the key residues including Thr26, Met49, Asn142, and Gln189, while Thr26, and His41 were found in hydrogen bond donor interactions with Compound 5 with a docking score of −10.7164. It has recently been demonstrated that ivermectin inhibits SARS-CoV-2 by up to 5000-fold *in vitro* with an IC 50 value of ∼ 2 μM (Jan et al., 2021; Kaur et al., 2021). In the docking study, ivermectin was selected as a standard reference inhibitor. The interaction details for the control compound are listed in Figure 7H. The control compound forms 05 hydrogen bonds with the key catalytic residues of main protease Asn119, Cys145, and Met165. The co-crystallized ligand (PDB ID; 6LU7) was removed from the active site and re-docked into thde binding site of 3CL^PR^ in order to evaluate the precision of MOE-Dock. The RMSD value between the top-ranked docked conformation and the co-crystallized ligand was 0.6532 (Figure S3), indicating the strong accuracy of the MOE-Dock procedure (Wadood et al., 2022c).

**TABLE 5:** Docking score and interaction of top five hits against the 3CL^pro^.

C. No	Docking score	Ligand	Receptor	Residues	Interaction	Distance	Energy (kcal/mol)
Compound 1	−12.0321	O 4	SG	CYS 145	H-donor	4.06	−0.5
		O 8	SG	CYS 145	H-donor	4.04	−0.8
		O 14	OG	SER 46	H-donor	2.96	−0.6
		C 28	SD	MET 49	H-donor	3.89	−0.8
		O 2	NE2	HIS 41	H-acceptor	3.29	−0.7
		O 8	NE2	HIS 163	H-acceptor	3.05	−0.7
		O 9	NE2	HIS 163	H-acceptor	3.28	−1.8
		O 11	CA	LEU 141	H-acceptor	3.49	−0.6
		6-ring	CA	THR 25	π -H	4.07	−0.6
Compound 2	−11.4527	O 13	SG	CYS 145	H-donor	4.40	−0.7
		O 15	SD	MET 49	H-donor	3.84	−0.5
		O 18	O	THR 26	H-donor	2.86	−1.4
		O 21	OD1	ASN 142	H-donor	2.84	−0.6
		O 25	SD	MET 165	H-donor	3.60	−1.2
		O 12	NE2	HIS 41	H-acceptor	2.96	−0.8
		O 19	NE2	HIS 163	H-acceptor	3.07	−1.9
		6-ring	N	SER 46	π-H	4.24	−1.4
		6-ring	N	THR 90	π-H	4.33	−0.6
Compound 3	−11.2783	O 8	SD	MET 49	H-donor	3.79	−0.5
		O 22	SG	CYS 145	H-donor	3.19	−1.1
		C 26	OE1	GLN 189	H-donor	3.13	−0.9
		O 22	NE2	HIS 41	H-acceptor	3.15	−1.0
		O 23	NE2	HIS 163	H-acceptor	3.19	−1.0
		6-ring	CA	LEU 141	π-H	3.80	−0.5
Compound 4	−10.9628	O 4	O	THR 26	H-donor	2.80	−2.2
		O 6	ND1	HIS 164	H-donor	2.95	−1.8
		O 9	OG1	THR 25	H-donor	3.05	−1.6
		C 13	SD	MET 49	H-donor	3.81	−0.6
		O 5	N	GLY 143	H-acceptor	3.16	−2.7
		6-ring	5-ring	HIS 41	π-π	3.27	−0.0
Compound 5	−10.7164	O 10	OD1	ASN 142	H-donor	3.11	−1.9
		O 15	O	GLN 189	H-donor	3.07	−1.0
		O 18	O	THR 26	H-donor	3.01	−1.8
		C 57	SD	MET 49	H-donor	3.94	−0.6
		O 18	N	THR 26	H-acceptor	2.95	−0.9
		O 30	NE2	HIS 41	H-acceptor	3.10	−0.6
IVERMECTIN	−9.5398	O 5	SG	CYS 145	H-donor	3.77	−0.6
		O 6	O	ASP 187	H-donor	2.91	−0.4
		C 35	SD	MET 165	H-donor	3.81	−0.5
		C 45	SD	MET 49	H-donor	3.49	−0.2
		O 13	ND2	ASN 119	H-acceptor	3.43	−0.6

### 3.6 MD simulation analysis

#### 3.6.1 Root means square deviation

**FIGURE 8:**
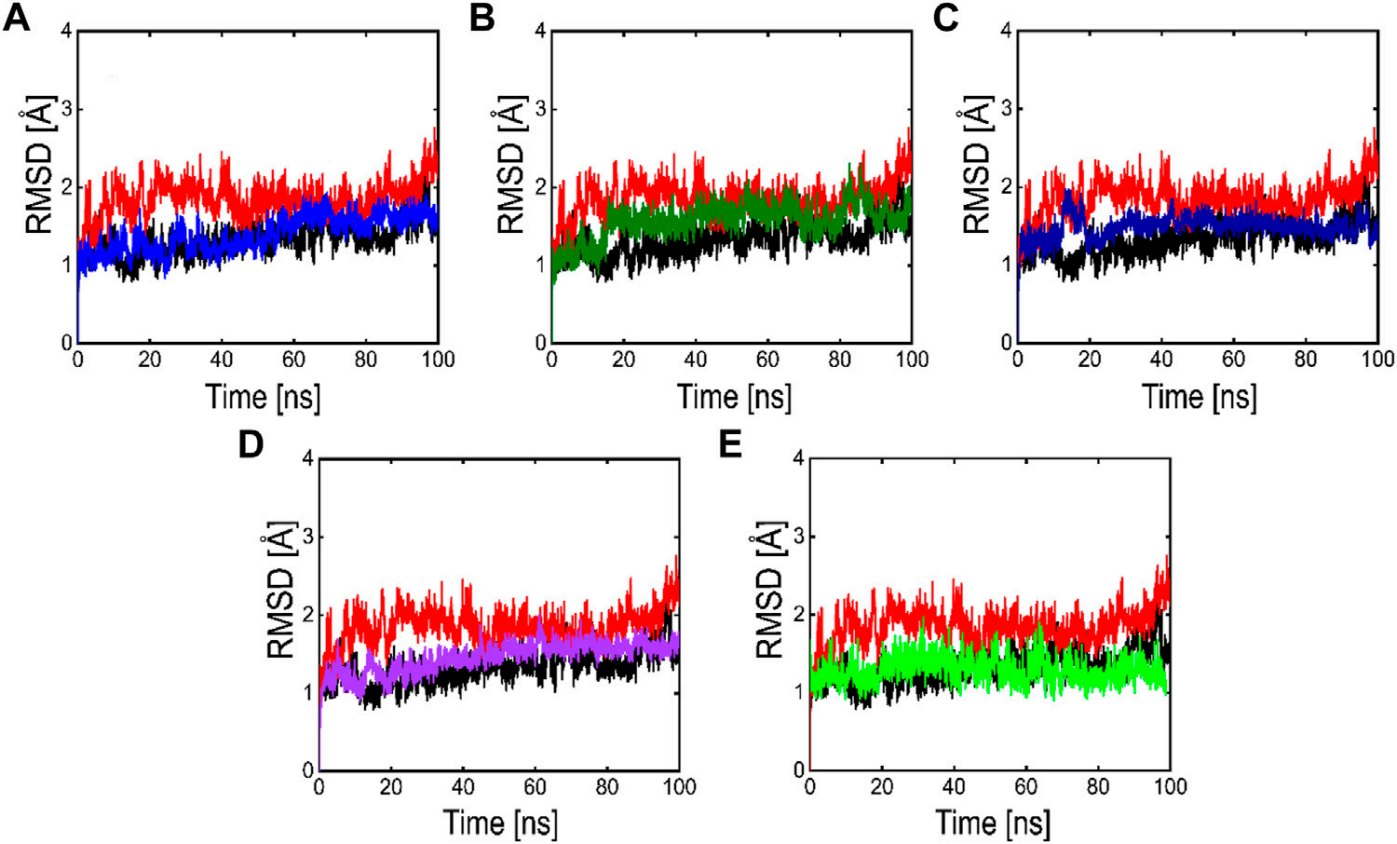
RMSD plots of the APO form (Black color), reference complex (Red color) and the top active phytochemicals **(A)** Compound 1 **(B)** Compound 2 **(C)** Compound 3 **(D)** Compound 4 and **(E)** Compound 5 bound to 3CL^PRO^.

Root means square deviation (RMSD) analysis was performed to calculate the stability of the top five phytochemicals and reference compound (ivermectin) in the active site of the main protease. We examined and compared the stability of these compounds with the reference and APO protein. The RMSD finding indicates that all these five phytochemicals show stable behavior but some minor deviation. For all the systems the averaged RMSD ranges between 1 and 3 Å. The average RMSD for ivermectin was initially 2.0 Å. Then a small increase was observed in RMSD up to 40 ns, soon after reaching 40ns the system acquired stability and remained stable for the rest of the simulation period. The complex Compound 1 shows significant stability as can be observed, however after 60 ns, the system briefly displayed a small variation. Then the system achieved stability and moved into the production phase. For Compound 2, RMSD reveals that the system shows highly stable behavior in the entire period of simulation, at 20ns minor fluctuations from its mean position were observed, afterward, the system gained stability and no more significant deviations were observed with the average RMSD value of 1.7 Å. For complex Compound 3, the system initially shows stable behavior, at around 15 ns a gradual increase in the RMSD curve was observed followed by a slight decrease in the RMSD curve at 20 ns. After that the system equilibrates with an average RMSD value of 2.1 Å as shown in Figure 8. The Compound 4 complex RMSD analysis reveals that the system initially shows an increase in the RMSD curve but soon after reaching 25 ns the system equilibrates and no significant fluctuations were observed for the rest of the simulation period which indicates the stable binding of Compound 4 compound in the catalytic pocket of 3CL^PRO^ with the average RMSD value of 1.4 Å. Afterward, we analyzed the RMSD of Compound 5 in the active site of 3CL^pro^, the RMSD curve of the corresponding complex has minor fluctuations at different time intervals, with an average RMSD value of 1.7 Å. The backbone RMSD for the phytochemical bound 3CL^PRO^ was slightly lower than the control indicating the stable binding of these phytochemicals which was further validated by RMSF and MM-GBSA analysis.

#### 3.6.2 Root mean square fluctuation

**FIGURE 9:**
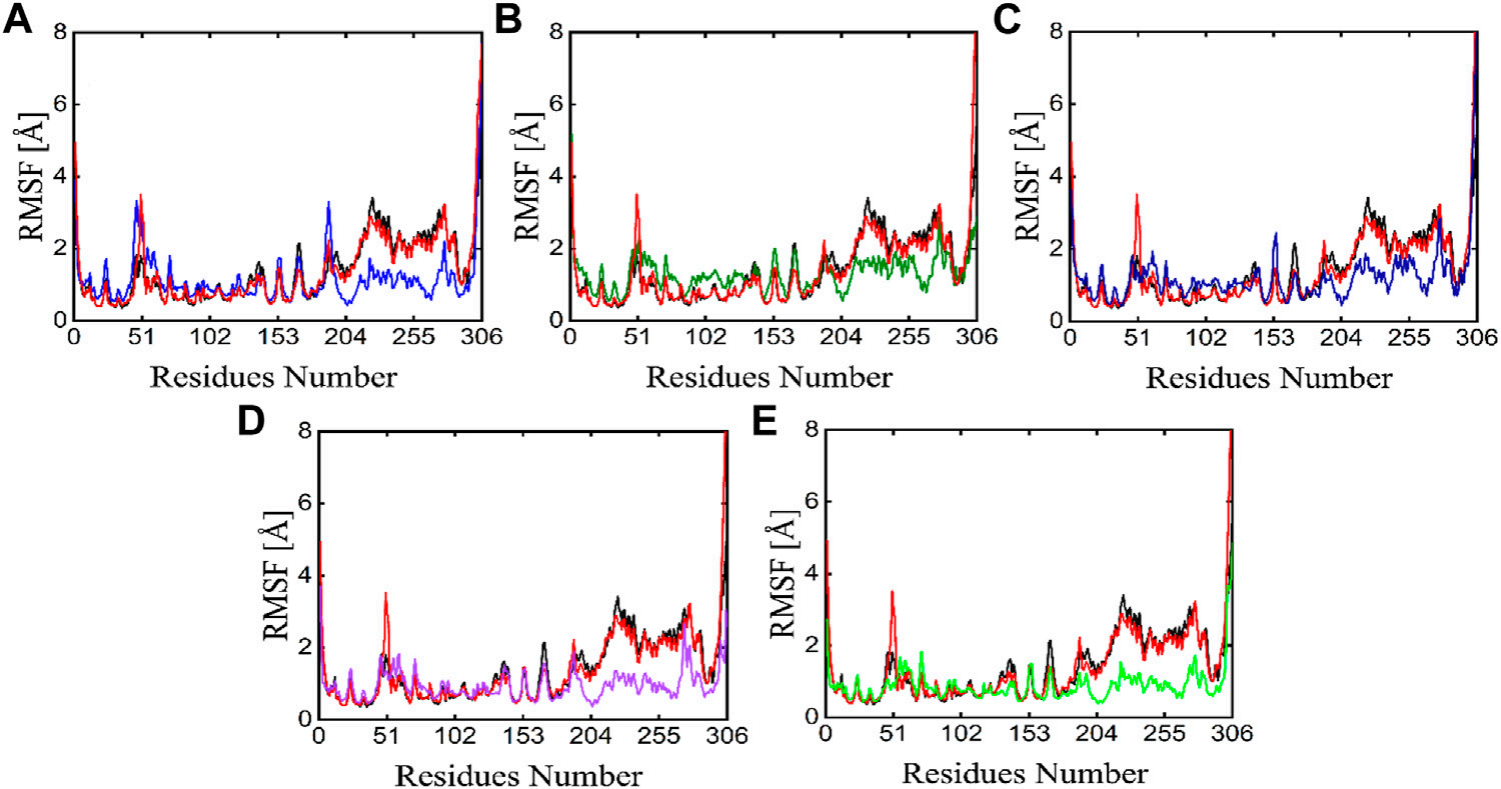
RMSF plots of the APO state (Black), control (Red) and the potent phytochemicals **(A)** Compound 1 **(B)** Compound 2 **(C)** Compound 3 **(D)** Compound 4 and **(E)** Compound 5.

The individual amino acid fluctuations of the main protease in complex with ligands were computed by RMSF analysis to assess the stability of the active site residues toward the compounds in the entire 100 ns MD trajectories (Figure 9). The RMSF of the main protease in the APO state, reference compound, and all five phytochemicals bounds to the main protease were analyzed and compared to each other, the black line in each plot represents the apo state while the red line indicated the residual flexibility of reference compound bounds to the target protein. Figure 9 shows that residues 51 and 250–260 show higher fluctuations. All these fluctuating residues were not found in the active site and these residues were far away from the active site indicating the stable binding of phytochemicals in the active site of the target protein.

#### 3.6.3 Radius of gyration

**FIGURE 10:**
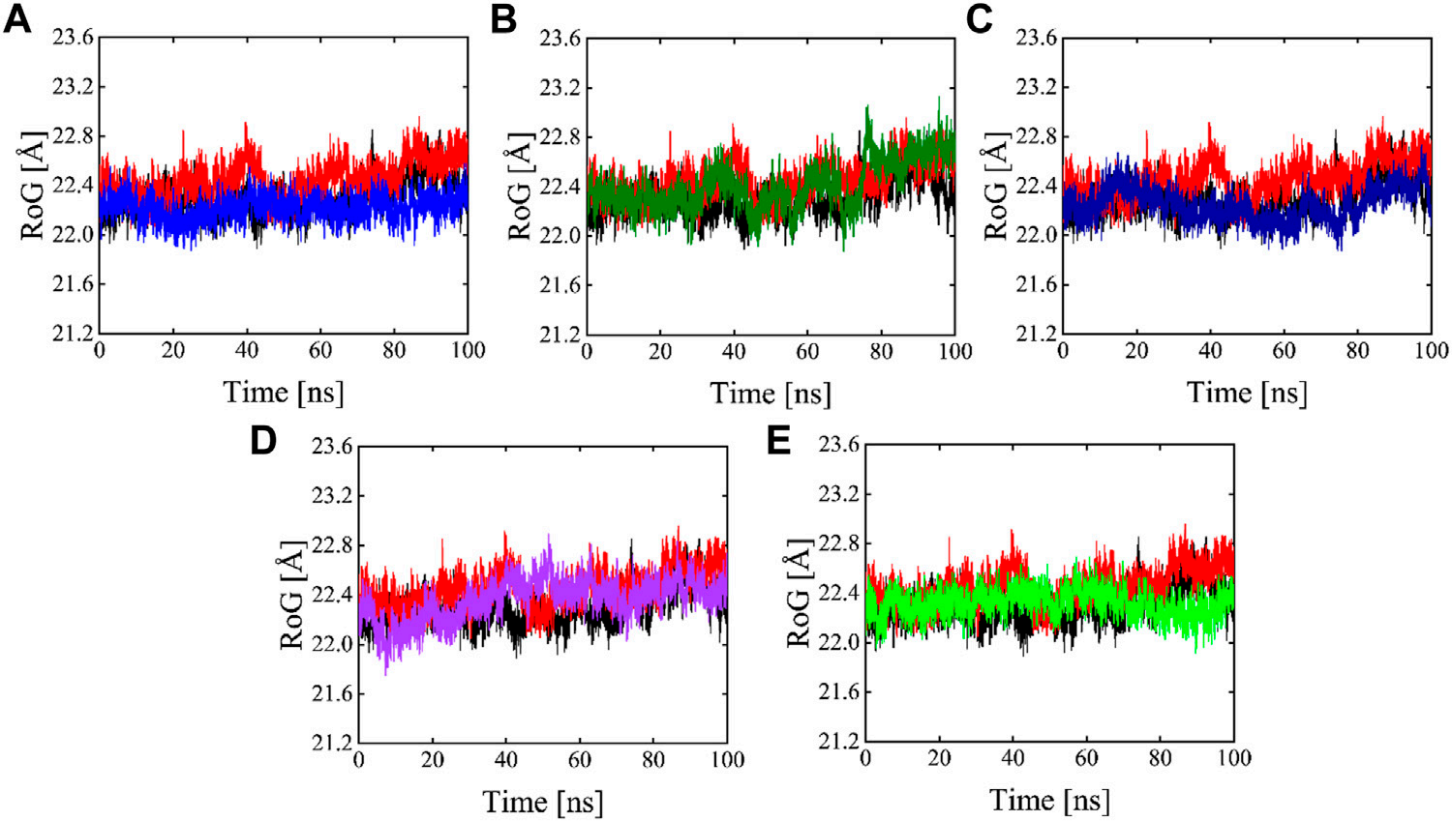
Rg plots of Apo (Black), red (reference), and Compound 1-5 are labeled different colors as **(A–E)** Respectively.

The radius of gyration is useful for exploring the compactness and folding of the protein, Higher Rg values are indicative of less compactness (more unfolded), while lower Rg values indicate more structural rigidity and strong compactness. The MD simulation study serves to illustrate the effects of inhibitors binding upon the conformation of protein molecules. As illustrated in Figure 10 the results of Rg analysis indicate that these phytochemicals bound to 3CL^PRO^ have less radius of gyration values compared to the apo state, which demonstrates the 3CL^PRO^, stability, and compactness after ligand binding. The reference compound, Compound 1, and Compound 4 have almost similar Rg values, with an average Rg value of 22–22.3 and 22–22.4 Å while Compound 2, Compound 3, and Compound 5 showed an average gyration of 22–22.5, 22–23.3 and 22–22.4 Å respectively. The compactness of the protein was significantly affected by the binding and unbinding of these phytochemical inhibitors.

#### 3.6.4 Dynamic cross-correlation matrix (DCCM) analysis

**FIGURE 11:**
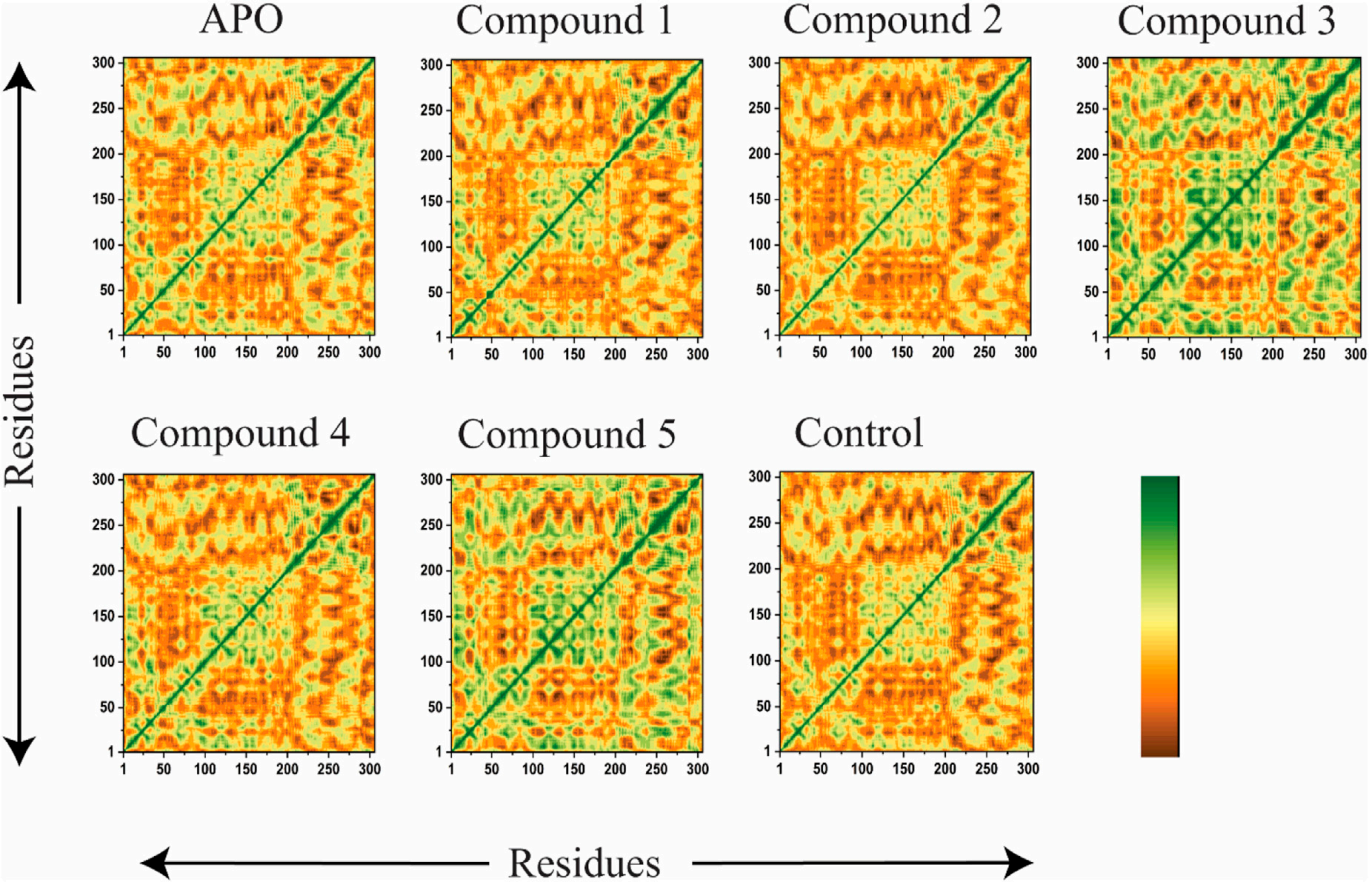
DCCM of the APO state, Compound 1, Compound 2, Compound 3, Compound 4, Compound 5, and ivermectin (control) bound to 3CL^PRO^. The positively correlated movement is represented by green color, while negatively correlated motion is indicated by deep brown color.

The extent of correlation motion between the residues imposed by the binding of compounds in the active site of 3CLpro was elucidated by the inter-residue correlation analysis. The results indicate that compound 1 in complex with the receptor active site residues showed significantly stronger parallel correlations motions in comparison with the control compound, which further validates that these positive correlation motions may be induced by the acquired interaction of these compounds with the key residues (25–50, 141–145,163), like hydrophilic, hydrogen and hydrophobic. Overall, the DCCM findings demonstrate that the control compound and our identified compound displayed comparable patterns of highly positive correlation. Furthermore, for compound 3 and compound 5 the nearby loops regions were also found in strong positive correlations as shown in Figure 11. The dark green color demonstrates a positive correlation in residues of protein while the dense brown color indicates a negative correlation between the protein residues. The negatively correlated residues move in an anti-parallel direction while the positively correlated residues move in a parallel direction.

### 3.7 GBSA results

#### 3.7.1 MM-GBSA analysis

Protein-ligand complexes from the MD simulation trajectories were used to calculate the energy parameters to assess the energetics of 3CL^PRO^ to the ligands. The binding free energies of each system were calculated using the MM-GBSA method. Table 6 display the computed average binding free energies and specific energetic contribution components of the final 500 frames. As can be observed, compound 1 has smaller free energy (−56.94 kcal/mol) followed by compound 2 (−55.65 kcal/mol), compound 3 (−53.58 kcal/mol), and compound 4 (−46.95 kcal/mol). It was observed that, as compared to the control system, all the ligands in complex with 3CL^PRO^ revealed high binding affinity demonstrating that all the systems are stable. Out of all, the binding affinity of system one was very high for the receptor. This outcome is consistent with the conclusion drawn from the earlier RMSD and docking analysis i.e., compound 1 showed stable dynamic behavior and established a greater number of non-covalent interactions (Figure 8A; Table 5).

**TABLE 6:** Represents MMGBSA Binding Free Energy (kcal/mol) calculation for the selected phytochemicals and control compound.

S. No	Compound name	VDWAALS	EEL	EGB	ESURF	-TΔS	DELTA TOTAL
1	Compound 1	−83.4745	−20.3304	56.6693	−9.8094	−18.4312	−56.9450
2	Compound 2	−79.3325	−20.6400	52.7843	−8.4635	−17.8254	−55.6517
3	Compound 3	−73.1537	−19.5693	51.8532	−8.5177	−19.2984	−53.5835
4	Compound 4	−64.4348	−16.3432	41.7462	−6.8571	−13.9835	−46.9500
5	Compound 5	−42.2227	−4.3191	13.2240	−4.7141	−10.8921	−38.0319
6	Ivermectin	−38.9027	−6.3834	20.7589	−4.3827	−14.5924	−28.9100

vdW = the van der Waals energy, EEL, electrostatic energy; ESURF, surface areas energy; EGB, the electrostatic contribution to the solvation free energy calculated by GB.

## 4 Discussion

The increased mortality rate of SARS-CoV-2 has created a pandemic situation globally, no effective drugs and treatments are available to treat COVID-19, however, many clinical trials are undergoing. New infectious agents, like SARS and MERS, have emerged in the last 20 years and have created epidemics. The functional significance of 3CL^pro^ in the viral life cycle and the lack of closely comparable human homologs make 3CL^pro^ an attractive target for the development of antiviral medications (Jin et al., 2020). By targeting the 3CL^pro^ most of the natural compounds play a significant role in the treatment of COVID-19 infections (Jin et al., 2020; Mengist et al., 2020). *In vitro*, animal models, and clinical trials are all used to study natural compounds that are extracted from medicinal plants, animals, and marine species for the treatment of COVID-19 (Wu et al., 2019; Wei et al., 2020; Sahoo et al., 2021). One of the most promising and effective strategies for combating the current pandemic is still seen to be the use of natural products (ying et al., 2001). Extractions from medicinal plants and their secondary metabolites frequently show strong antiviral properties. Some *in vitro* studies showed that PSM and viral incubation had direct interference. The viral protein, its lipid layers, and the cell’s lysis can be destroyed by the plants’ metabolites (Akram et al., 2018). There are about six to seven thousand different plant species in Pakistan, of which 700 are regularly used as medicines (Khan et al., 2022). The SARS CoV 2 RdRp was chosen as a receptor for computational drug development in the previous study in which 200 phytochemicals were used for virtual screening. The top 10 ligands among 200 total ligands were chosen based on drug discovery criteria such as S-score, ligand interactions, hydrophobic interactions, and drug-likeness (Mahrosh and Mustafa, 2021).

Developing a new drug against the virus is time-consuming and costly. The ability of computer-aided drug design, on the other hand, to screen a large library of small molecules quickly and accurately may help the researcher to develop a new therapeutic agent against SARS-CoV-2 (Wang, 2020). The virtual screening workflow has made it possible to screen the enormous, diverse chemical library for the identification of powerful inhibitors (Neves et al., 2018). In the drug development processes, machine learning (ML) techniques are frequently used to categorize compounds as potentially active or inactive against a given protein target (Patel et al., 2020). Structure and ligand-based virtual screening frequently yield a high proportion of false positive hits (Deng et al., 2015). To reduce the false positive hits in this work, we used to machine-learning-base virtual screening for the prediction of new inhibitors against the 3CL^pro^. K-nearest neighbor (KNN), support vector machine (SVM), and Random Forest (RF) algorithm three of the most popular ML algorithms were chosen for virtual screening workflow. In general, classifier performance is evaluated in terms of accuracy. KNN achieved 0.93% accuracy SVM achieved 0.96% accuracy, whereas RF produced 0.99% accuracy on the train set. Our results revealed the best performance of the RF model, so we used the RF model to classify the Asian phytochemicals. Out of 4,000 phytochemicals, a total of 26 phytochemicals were predicted as active against the 3CLpro. These active hits were further docked into the active site of the main protease. Among the 26 docked compounds, Compound 1 was found as the most potent with a docking score of −12.03 and it formed four H-donor interaction with CYS145, SER46, MET49, and four H-acceptor interactions with HIS41, HIS163, LEU141 one pi-H interaction with THR25 active site residues. Compound 2 was found as the second most potent hit with a docking score of −11.45 followed by Compound 3. Compound 2 formed a total of five hydrogen bonds donor interactions with the active site residues including CYS145, MET49, THR26, ASN142, MET165, and two H-acceptor interactions with HIS41, and HIS163. The docking scores as well as interactions of Compound 3, 4 and 5 were also good as compared to the standard compound. The docking score of reference compound ivermectin was −9.53 and it formed a total of four H-donor interactions with CYS 145, MET 165 and one H-acceptor interaction with ASN 119 active site residue. Additionally, dynamics simulation was carried out to comprehend and support the molecular docking study. For all the systems the averaged RMSD was found between 1 and 3 Å. The averaged RMSD for ivermectin was 2.0 Å, initially, up to 40 ns the system undergoes raised up in the RMSD value up to 40 ns, and soon after reaching 40 ns the system acquired stability and remained stable for the rest of the simulation period. The complex Compound 1 shows significant stability as can be observed, however after 60 ns, the system briefly displayed a tolerable variation. The system thereafter became stable and moved into the production phase. For Compound 2, the finding of the stability index in terms of RMSD reveals that the system shows highly stable behavior in the entire period of simulation, at 20 ns minor fluctuations from its mean position were observed, afterward, the system gained stability and no more significant deviations were observed with the average RMSD value of 1.7 Å. For complex Compound 3, the system initially shows invariant behavior, up to 15 ns a gradual increase in the RMSD curve was observed followed by a slight decrease in the RMSD curve at 20 ns afterward the system attains the equilibrated with the averaged RMSD value of 2.1 Å. The protein structure’s compactness as a function of time can be evaluated by the radius of gyration (Ajmal et al., 2022). The RoG analysis revealed compound 1, and compound 4 have almost similar Rg values, with an average Rg value of 22–22.3 and 22–22.4 Å while compound 2, compound 3, and compound 5 showed an average gyration of 22–22.5, 22–23.3 and 22–22.4 Å respectively. The Rg analysis of all the simulated complexes revealed that these phytochemicals formed stable and compact complexes with 3CL^PRO^. All the short-listed phytochemicals revealed good binding affinity for 3CL^PRO^. Compound 1 has smaller free energy (−56.94 kcal/mol) followed by compound 2 (−55.65 kcal/mol), compound 3 (−53.58 kcal/mol), and compound 4 (−46.95 kcal/mol). It was observed that, as compared to the control system, all the ligands in complex with 3CL^PRO^ revealed high binding affinity demonstrating that all the systems are stable. The RMSF analysis revealed that Domain II had a stable behavior, whereas Domain I and Domain III’s amino acid residues had more flexibility in the helix and turn regions. The overall finding of RMSD and binding energy indicates that our novel phytochemicals have higher binding capacity toward the active site of the main protease. ML-based workflow combined with molecular docking and molecular dynamics approach reveals that the predicted new active phytochemicals may disrupt the SARS-CoV-2 3CL^pro^ function.

## 5 Conclusion

We used *in silico* machine learning tools for drug designing against the SARS-CoV-2 3CL^pro^. The phytochemical dataset with more than 4,000 chemicals derived from the PubChem database was used for virtual screening against 3CL^pro^. Furthermore, the compound’s inhibitory potential was explored using the molecular docking and MD simulation study. Using these advanced approaches, we found high-potential therapeutic compounds that can possibly inhibit SARS-CoV-2 pathogenesis. The virtual screening process, which includes MM-GBSA methods assists in reducing the list from over 4,000 possible lead compounds to 26 compounds. This research relies only on various computational tools and further it is recommended to evaluate the *in-vitro* inhibitory potential of these short-listed compounds. Successful assessment and *in vitro* evaluation of these compounds will help us to use them as a therapeutic option to treat and cope with COVID-19.

## Data Availability

Data will be provided upon reasonable request from the corresponding author of this manuscript. Requests to access these datasets should be directed to awadood@awkum.edu.pk.
