# Supplementary material for: Identification of novel inhibitors for SARS-CoV-2 as therapeutic options using machine learning-based virtual screening, molecular docking and MD simulation
Source: Front Mol Biosci. 2023 Mar 7;10:1060076. doi: 10.3389/fmolb.2023.1060076 (PMC10028080; doi:10.3389/fmolb.2023.1060076)
Supplement: Supplementary file 1 [file Table2.DOCX]

**Identification of Novel Inhibitors for SARS-CoV-2 as Therapeutic Options using Machine learning-based Virtual Screening, Molecular Docking and MD Simulation**

Abdus Samad 1, Amar Ajmal1, Beenish Khurshid1, Arif Mahmood2,3, Ping Li^4^, Syed Mansoor Jan^1^, Ashfaq Ur Rehman5, Ashraf N. Abdalla^6,^ and Abdul Wadood1*

1. Department of Biochemistry, Abdul Wali Khan University, Mardan, KPK, Pakistan
2. Center for Medical Genetics and Hunan Key Laboratory of Medical Genetics, School of Life Sciences, Central South University, Changsha, 410078, Hunan, China
3. Institute of Molecular Precision Medicine, Xiangya Hospital, Central South University, Changsha, Hunan, China
4. Institutes of Biomedical Sciences, Shanxi university, Taiyuan, China
5. Department of Molecular Biology and Biochemistry, University of California Irvine, CA 92697-3900 United States
6. Department of Pharmacology and Toxicology, College of Pharmacy, Umm Al-Qura University, Makkah 21955, Saudi Arabia. anabdrabo@uqu.edu.sa

**Table S2.** ADMET (Absorption Distribution Metabolism Excretion Toxicity) properties of the final hits

| Compound | toxic | Weight(g/mol) | TPSA | logP | logD | logS |
| --- | --- | --- | --- | --- | --- | --- |
| 91895373 | no | 652.65 | 223.29 | 0.20 | 0.28 | -3.29 |
| 10606127 | no | 1078.98 | 426.73 | -1.34 | -1.19 | -4.59 |
| 5318857 | no | 943.09 | 304.21 | -0.39 | -0.39 | -4.00 |
| 457885 | no | 498.48 | 177.14 | 2.15 | 4.16 | -4.16 |
| 44256914 | no | 1390.24 | 530.02 | 1.22 | 1.89 | -8.28 |
| Reference | no | 875.11 | 170.06 | 2.10 | 2.10 | -6.26 |


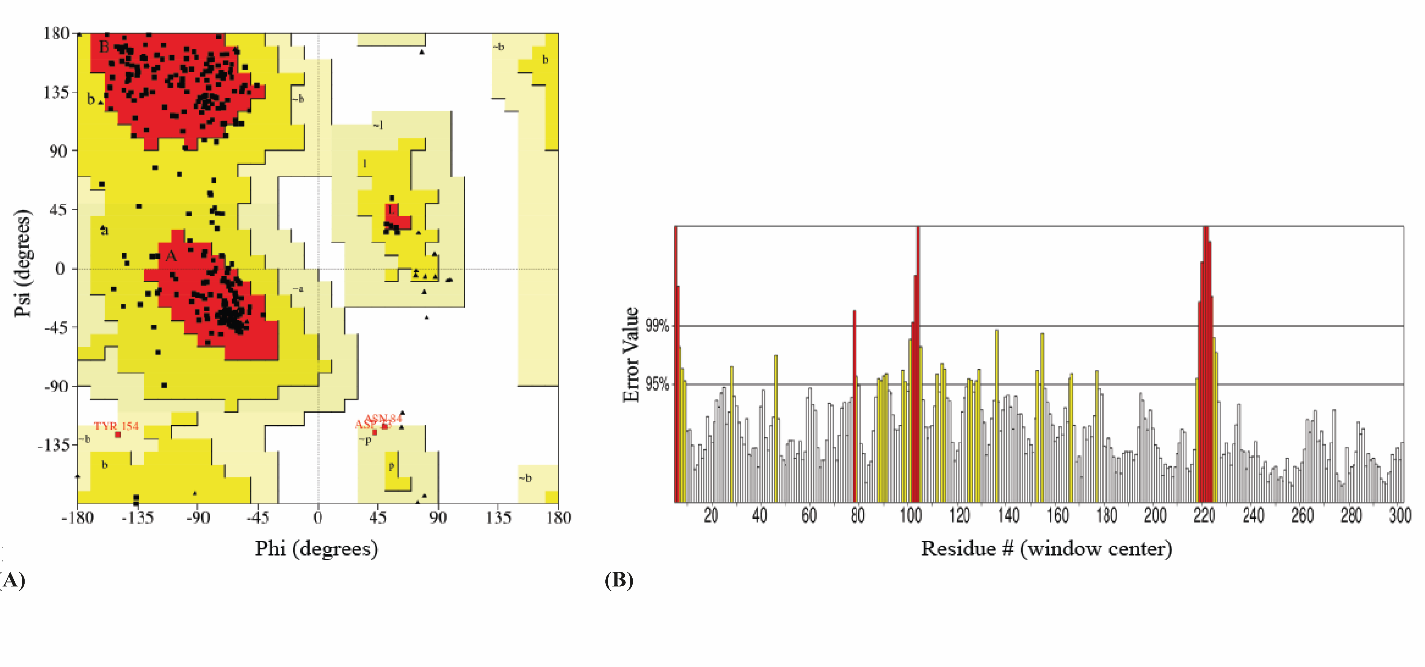


**Figure S1**. Validation of the 3C-like protease (PDB ID, 6LU7). A) The Ramachandran plot for the 3CL^PRO^ structure showed that 84.5% of residues were in the most favored region, while 14.3% were in the additional allowed region, 1.1% residues were in the generously allowed region and 0% residues were in the disallowed region demonstrating the high quality of the 3CL^PRO^ structure. B) The ERRAT server predicted an overall quality factor of 85.90 for the 3CL^PRO^ structure used in our study


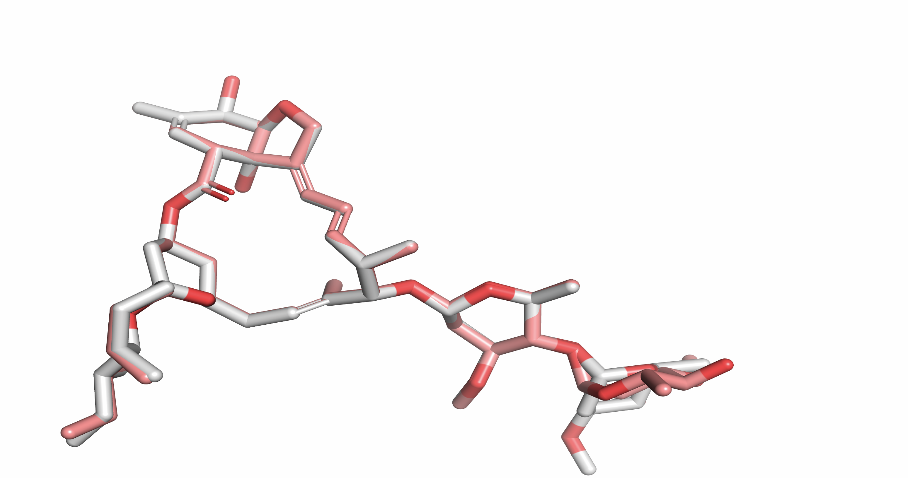


**Figure S2.** Superposition of original pose and docked co-crystalline ligand. The red color represents the native co-crystallized ligand, and the silver color is the docked ligand.


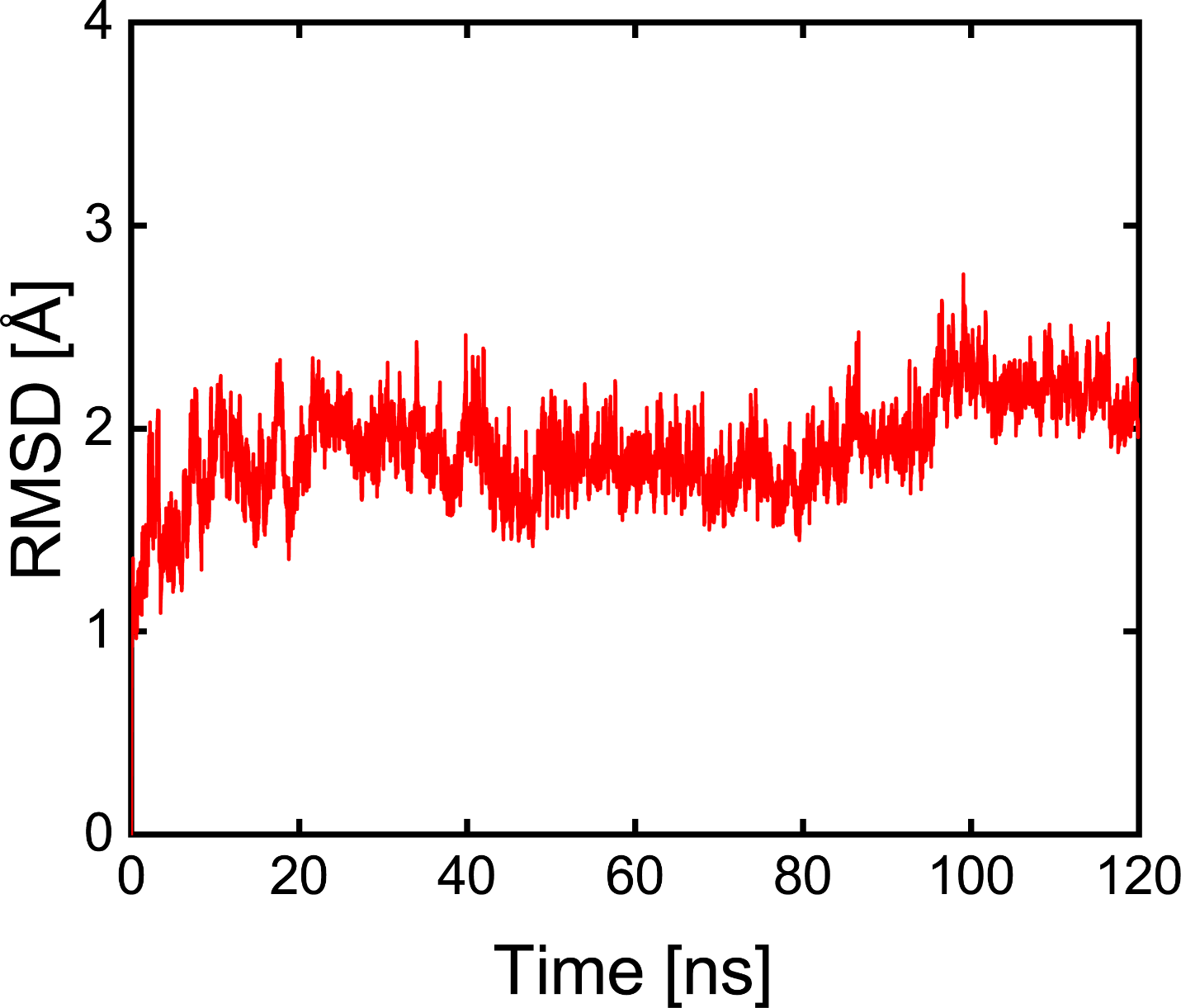


**Figure S3.** RMSD curve of the reference compound.
